# Development of a New Type of Flame Retarded Biocomposite Reinforced with a Biocarbon/Basalt Fiber System: A Comparative Study between Poly(lactic Acid) and Polypropylene

**DOI:** 10.3390/polym14194086

**Published:** 2022-09-29

**Authors:** Jacek Andrzejewski, Sławomir Michałowski

**Affiliations:** 1 Institute of Materials Technology, Faculty of Mechanical Engineering, Poznan University of Technology, Piotrowo 3 Stree, 61-138 Poznan, Poland; 2 Department of Chemistry and Technology of Polymers, Cracow University of Technology, 24 Warszawska Street, 31-155 Kraków, Poland

**Keywords:** poly(lactic acid), polypropylene, flame retardancy, mechanical performance, sustainable fillers, injection molding

## Abstract

A new type of partially biobased reinforcing filler system was developed in order to be used as a flame retardant for polylactic acid (PLA) and polypropylene (PP)-based composites. The prepared materials intended for injection technique processing were melt blended using the novel system containing ammonium polyphosphate (EX), biocarbon (BC), and basalt fibers (BF). All of the prepared samples were subjected to a detailed analysis. The main criterion was the flammability of composites. For PLA-based composites, the flammability was significantly reduced, up to V-0 class. The properties of PLA/EX/BC and PLA/EX/(BC-BF) composites were characterized by their improved mechanical properties. The conducted analysis indicates that the key factor supporting the effectiveness of EX flame retardants is the addition of BC, while the use of BF alone increases the flammability of the samples to the reference level. The results indicate that the developed materials can be easily applied in industrial practice as effective and sustainable flame retardants.

## 1. Introduction

The flammability of polymer-based materials is one of the essential restrictions on the use of plastics. Plastic parts are usually easy to ignite and once ignited, the flammable gases, fumes, and decomposition products can fuel the process of further burning. For that reason, the modification of polymer-based materials is challenging; in practice, it is not possible to obtain fireproof plastics. However, modifications of the material are aimed to slow down or limit this combustion process and smoke emission, so as to reduce the hazardous effects of the flammability [1,2]. 

Even for composite materials, where thermoset or thermoplastic resin is used as a binder for mineral fillers, flammability is considered a serious problem. Due to the high thermo-mechanical resistance of reinforced composites, the area of application for this type of material frequently includes electrotechnical devices and machines operating at elevated temperatures. In such cases, the risks associated with fire ignition are very high. Despite these threats, manufacturers more often shy away from the use of metal composites in favor of composite products, especially those obtained by an injection molding technique. The use of flammability limitation techniques for this type of product is, therefore, a necessity. For many years, the most popular flame retardants used in thermoplastics have been halogen-based additives. For these compounds, the flammability is limited by the formation of free radicals. At high temperatures, these highly reactive compounds scavenge the polymer degradation products. Despite the increased effectiveness of halogenated-based agents, their use is now prohibited mainly due to the hazardous nature of these compounds, especially their toxicity and negative environmental impact [3,4,5,6]. Other flame retardants are still in common use, the most popular being metallic hydroxides (MH) and phosphorus-based additives. In the first category of MH materials, the most prevalent compounds are aluminum tri-hydroxide (ATH) and magnesium di-hydroxide (MDH). In both cases, the flame-retardant mechanism is based on water vapor release, which happens during combustion. Unfortunately, for this type of compound, the visible reduction in the flammability of polymers occurs with a minimum content of 50 wt%, which always leads to the deterioration of most of the essential material properties.

Currently, the most promising group of polymer flame-retardants are phosphorous-based compounds due to their lack of toxicity and relatively high effectiveness. For these same reasons, materials of this type were used in this research cycle. The two most popular phosphorous-based flame-retardants are red phosphorous and ammonium polyphosphate (APP). The flame-retardancy mechanism for this type of compound is based on the decomposition of the APP fillers into phosphoric acid and transformation into polyphosphoric acid at higher temperatures, leading to the formation of a rigid char layer. The initial decomposition reaction takes place in oxygen or nitrogen, which means that oxygen/nitrogen-containing plastics are more favorable for modification. In the research discussed in this article, flame retardants were used for a PLA and PP matrix; it was expected, due to the presence of oxygen in the PLA structure, that the effectiveness of flame retardancy for this polymer should be higher.

In the bio-based or biodegradable-plastics category, research on the use of flame retardants has mainly focused on PLA and its composites. At present, the study on PLA flame retardancy covers the use of metal hydroxides [7,8,9], carbon/graphite-based fillers [10,11,12,13], and nano additives [10,14,15,16]. The use of phosphorus-based compounds is also widely described in the literature and applies not only to pure PLA [17,18,19,20] but also to other types of biodegradable materials such as poly butylene succinate PBS [21,22], thermoplastic starch TPS [23,24], and different kinds of blends [25,26]. The necessity to use flame retardants is essential for composites with the addition of natural fillers. For this reason, the polymer matrix is very often made of traditional thermoplastics such as PVC, PE, or PP. The category of wood-polymer composite (WPC) materials is particularly important as the content of wood fillers usually exceeds 50 wt%. These composites are often used in construction; therefore, the appropriate selection of the matrix/filler/flame retardant system is the key to obtaining low flammability [27,28,29,30]. Since the total amount of fillers during the extrusion processing of WPC profiles is usually very high, it is possible to obtain the desired mechanical properties. However, the injection molding of high filler content composites is more difficult due to the need to maintain a sufficiently low viscosity of the material. This forces the use of more efficient reinforcement systems, usually based on glass fibers. For the presented study, basalt fibers were used, which, in addition to more sustainable manufacturing methods, are also characterized by higher thermal resistance than standard E-glass fibers [31,32,33].

For several years, the use of biochar/biocarbon particles as polymer filler has begun to be not only the subject of research work but also a competitive technology in many industrial applications [34,35]. There are already known examples of applications in the packaging and automotive industries, where, in many cases, biocarbon has replaced mineral fillers [36,37,38]. So far, the use of biocarbon fillers has mainly been considered a method of improving the ecological balance of polymer products. It has a logical justification in the case of products manufactured for the packaging industry or other types of products with low strength requirements [39,40]. For more demanding applications, in particular, in the technical applications of composites, the use of biocarbon has many limitations related to the low reinforcement factor for popular thermoplastics such as PP or PE in particular [41,42,43,44,45]; some exceptions are nylon plastics, where the high polarity of the polymer structure improves the effectiveness of matrix-filler interactions [46,47,48,49,50]. For the presented research, the use of bio-carbon fillers was aimed at limiting the flammability of the developed composites. Usually, materials of this type, due to the high addition of fillers, lose many desirable mechanical properties, therefore, the hybridization of the structure with synthetic fibers is usually a necessary procedure [51,52,53].

The idea of using biocarbon/biochar as a helpful material in reducing the flammability of polymer materials has not been a common research topic. Preliminary work on this subject was carried out by Snowdon et al. [54], however, flammability was not the core subject of the research. Another promising study was presented by Li et al. [55] where biocarbon modified with chitosan was used in the modification of PLA. Another study on the use of biocarbon/ATH mixture was presented by Wang et al. [56].

The aim of our study was to assess the possibility of using the hybrid filler system containing biocarbon (BC) particles, APP, and basalt fibers (BF) for comparative purposes. Due to the fact that both BC and BF belong to a group of sustainable materials, we decided to use PLA as the main investigated polymer, while polypropylene (PP) was used for comparative purposes. Since APP flame-retardants are most effective for oxygen-containing polymers, the expected results for PLA are expected to be more favorable. The developed materials are intended to be manufactured using injection molding technology; therefore, during the study, the materials were subjected to a two-stage processing procedure involving twin-screw extrusion blending and injection molding. The obtained samples were tested using several measuring methods. Mechanical properties were evaluated by static tensile/flexural tests and Charpy impact resistance measurements. Thermal analysis was carried out using the thermogravimetric (TGA) method and differential scanning calorimetry (DSC) analysis. Thermo-mechanical properties were compared using DMTA analysis and HDT/Vicat tests. Finally, flammability was evaluated using the microcalorimetry analysis and UL-94 method.

## 2. Materials and Methods

### 2.1. Materials

The type of poly(lactic acid) used during this study was Ingeo PLA 3001D, from NatureWorks (Minnetonka, MN, USA). The polypropylene resin was Moplen PP HP500N, from LyondellBasell (Plock, Poland). For all flame retarded samples, we used ammonium polyphosphate (APP); the filler was a supplier in powdery form. The type used was Exolit AP 422 (from Clariant, Muttenz, Switzerland). For reinforcement, we used chopped basalt fibers (BF) type BCS 13-1/4”-KV02M from Kamenny Vek company (Dubna, Russia). The biobased filler was biocarbon powder BC, obtained from the pyrolysis of the wood chips. The raw filler was supplied by the Fluid company (Sedziszow, Poland). Before processing, the BC filler was ball milled; the whole procedure and properties of the filler are presented in our previous studies [49,57]. The basic properties of all polymers and fillers are listed in Table 1. Data were collected from technical data sheets. 

### 2.2. Sample Preparation

All materials formulations were melt mixed using a twin-screw extruder. The machine type used was Zamak EH-16.2D (Zamak Merkator company, Skawina, Poland), equipped with co-rotating 16 mm screws. Before processing, both polymers were dried in a cabinet oven to avoid unnecessary moisture (at 60 °C for 6 h). Powdery fillers (EX and BC) were also dried (80 °C/12 h), while BF fibers were used as received. The melt blending of all materials was conducted using the same conditions. All ingredients were dry-blended before extrusion. The extrusion temperature measured at the die-head was set to 195 °C, while the screw speed was 100 rpm. The extruded material was transferred to the pelletizer using the air-cooled belt conveyor. The obtained pellets were collected in sealed bags, while before injection molding, the drying procedure was again applied (60 °C, 6 h). For the purpose of sample shaping, we used the electric injection molding press model Engel E-MAC 50 (from Engel GmbH, Schwertberg, Austria). We used the same procedure for both types of materials (PLA and PP-based). The injection molding temperature was 210 °C (measured at the nozzle), and the mold temperature was set to 30 °C. The injection/holding pressure was 800/400 bar, respectively, while the holding/cooling time was 10/30 s. The obtained rectangular bars and dumbbell samples were collected for conditioning (at least 48 h). The full list of samples is highlighted in Table 2.

### 2.3. Characterization

The mechanical properties of the obtained materials were evaluated using static tests (tensile, flexural) and a notched Izod impact test. The tensile test was performed in accordance with the ISO 527 standard, with measurements conducted with a cross-head speed of 10 mm/min. Flexural tests (ISO 178) were performed at the rate of 2 mm/min with a span distance of 64 m. Tensile/flexural measurements were conducted using the Zwick/Roell Z010 universal testing machine. Izod impact resistance tests (according to ISO 180 standard) were performed on the notched samples where the notch depth was 2 mm. We used the Zwick/Roell HIT 15 machine with a hammer equipped with a 5 J energy pendulum.

A scanning electron microscope (SEM) analysis was used to evaluate the structure’s appearance. The observed samples were cryofractured; the specimen was immersed in liquid nitrogen before breaking. Scanning observations were conducted on the surface covered with a conductive layer of gold using a sputter coater. An EVO 40 SEM microscope (Carl Zeiss AG, Jena, Germany) was used to conduct the main analysis.

Differential scanning calorimetry (DSC) measurements were performed using the standard heating/cooling/heating procedure. Tests were carried out from 20 to 230 °C at the heating/cooling rate of 10 °C/min. During the measurements, samples (≈5 mg) were placed inside the aluminum crucible; the oven chamber was purged with nitrogen (N2 flow = 20 mL/min), and we used the pierced lid. Samples were also cut from the injection-molded specimens. The apparatus used was the DSC F1 Phoenix from Netzsch company (Selb, Germany).

Thermogravimetric tests (TGA) were conducted using the Libra 209 F1 apparatus from the Netzsch company (Selb, Germany). The measurements were conducted from 30 to 800 °C at a heating rate of 10 °C/min. Similar to DSC, all samples were tested under a protective atmosphere of nitrogen. The average size of the samples was around 10 mg. 

Thermal analysis was supplemented with dynamic mechanical and thermal analysis (DMTA) measurements. For the purpose of the study, the rotational rheometer Anton Paar MCR301 was used. The apparatus was equipped with solid sample clamps; we used the torsion mode deformation. All tests were conducted under the same conditions using rectangular samples (50 × 10 × 4 mm). The temperature range was set to 25–150 °C, while the heating rate was 2 °C/min. Constant strain amplitude was equal to 0.01%, while the deformation frequency was 1 Hz. The results in the form of storage modulus and tan δ plots were collected. 

Flammability tests of the prepared samples were conducted using the pyrolysis combustion flow calorimeter (PCFC). Tests were carried out in accordance with the ASTM D7309-2007 standard. A microcalorimeter is a device designed to determine the rate of heat release by materials. The test method is based on the measurement of heat and oxygen loss during the thermal decomposition of a small sample of the material. The decomposition is carried out in an inert gas atmosphere in the temperature range of 150–750 °C with a heating rate of 1 °C/s. Then, the pyrolysis gases are oxidized in a high-temperature furnace at a temperature of 900 °C for complete oxidation. The results obtained during the tests allow for the determination of the HRR and THR parameters as well as the time and temperature of the HRR peaks.

Standard flammability tests were carried out using the UL-94 classification. Horizontal (HB) and vertical (V) combustion method measurements were performed using samples with dimensions of 125 × 10 × 4 mm, according to the PN-EN 60695-11-10 standard. Burn time was measured, and the dripping of burning material was monitored. According to the above observations, the combustion index, V, of the samples was calculated; it was on this basis that the materials were classified (according to the UL 94 HB methodology).

## 3. Results and Discussion

### 3.1. Structure Evaluation—Scanning Electron Microscopy Observations

**Figure 1:**
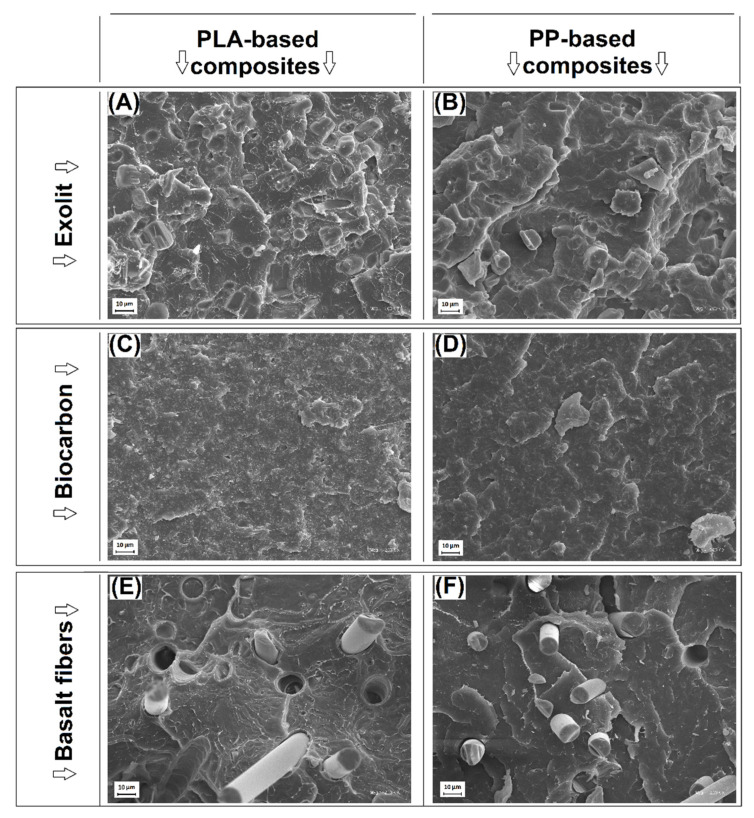
The structure appearance of the (**A**,**B**) EX-based composites, (**C**,**D**) BC-based, and (**E**,**F**) BF-based materials.

**Figure 2:**
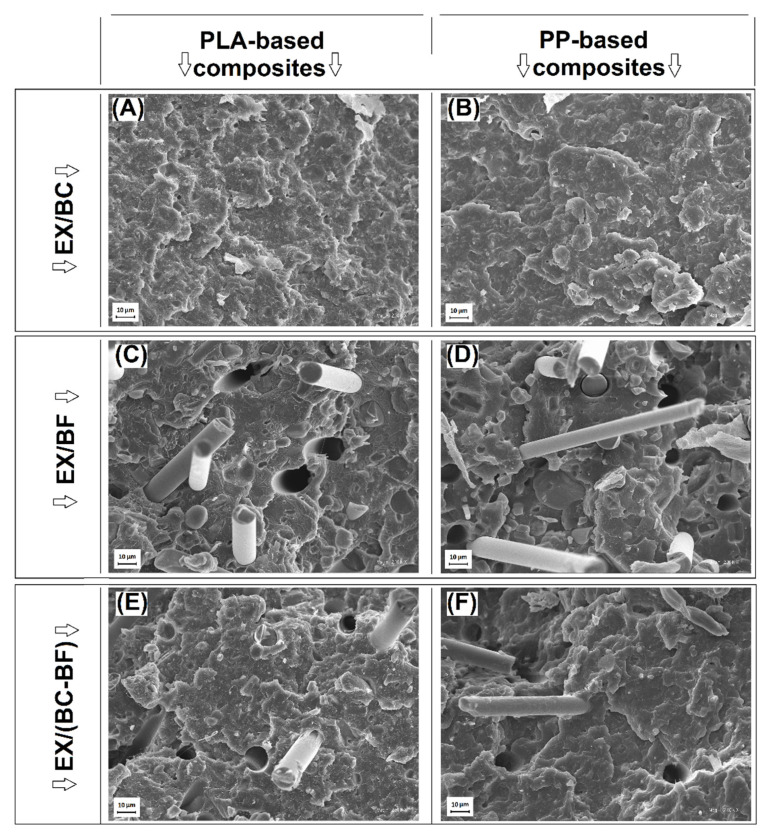
The structure appearance of the hybrid samples: (**A**,**B**) EX/BC, (**C**,**D**) EX/BF, and (**E**,**F**) EX/(BC-BF).

The structure appearance of EX, BC, and BF-based samples is presented in Figure 1, while the pictures presenting the structure of the hybrid samples are shown in Figure 2. The direct comparison between the individual filler types revealed large differences in the structure appearance. The structure of composites with the addition of basalt fibers shows a rather typical picture of composites reinforced with short fibers [58,59]. It is worth noting that the structure appearance for both PP and PLA suggests a lack of strong matrix-fiber interactions since the composite interface can be clearly distinguished. A more interesting observation can be made when comparing the structure of powder filler composites EX and BC. For EX-based composites, the structure observations revealed a large number of irregular ammonium phosphate particles, with the average particle size around 20 µm, similar to other studies [60]. The particle size for the BC filler is much smaller (≈1 µm), which is confirmed by SEM analysis [49]. The morphology of the sample surface revealed the brittle nature of the fracture mechanism. The irregular surface of the sample with the addition of EX is due to the presence of a relatively large filler particle rather than a more complex crack path. Since the structure of BC-based samples revealed that the dispersion of the BC particles was very uniform for both types of polymers, the expected mechanical properties should be more favorable than for EX-based samples, especially considering the significantly smaller size of the filler particle size for BC. 

The main part of the research was focused on the examination of the composites with the mixed filler system, where, for all materials, the main filler was EX flame retardant (Figure 1A) while BC, BF, and BC/BF fillers were used as additional/supporting additives. The structure appearance for EX/BC composites was quite similar to pure EX materials since the large EX particles are the dominant element of the sample images. The presence of the BC filler was also visible; however, due to the smaller size of the BC particles, they are less distinctive. The addition of BF into the structure of EX/BF and EX/(BC–BF) samples led to the formation of regular holes, which are the residue of the fibers. The random orientation of the fibers suggests the reduction in anisotropy in the arrangement of the fibers during the injection process. That phenomenon was observed in our previous studies as well [57,61]. Spherical particles were still visible for all samples reinforced with short BF. Interestingly, the was no visible difference between the PP and PLA samples. For both polymers, the matrix structure is characterized by a slightly rough fracture surface, which is a typical feature for partly crystalline polymers. 

### 3.2. Thermal Stability—Thermogravimetric Analysis (TGA)

**Figure 3:**
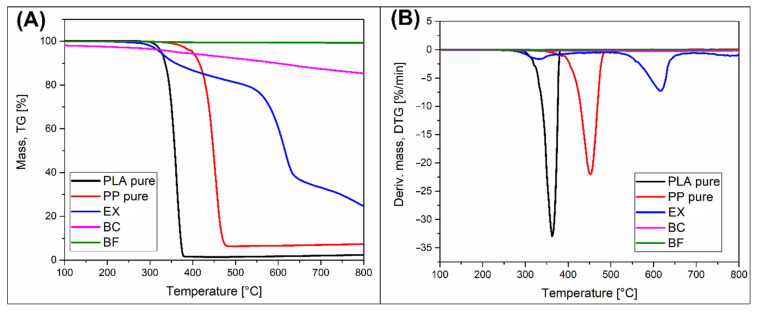
The thermogravimetric (TGA) analysis of pure polymers and filers: (**A**) TG and (**B**) DTG plots.

**Figure 4:**
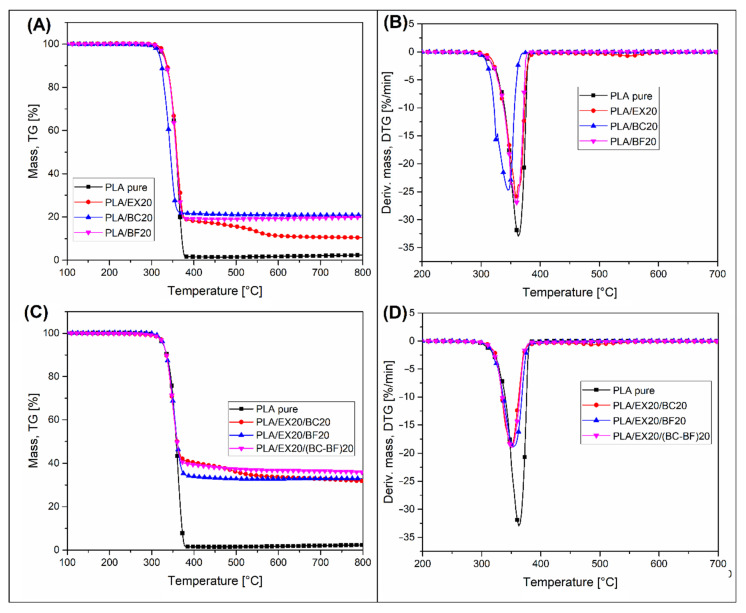
The thermogravimetric (TGA) analysis of PLA-based samples. Results are presented in the form of weight loss TG (**A**,**C**) and derivative weight loss plots DTG (**B**,**D**).

**Figure 5:**
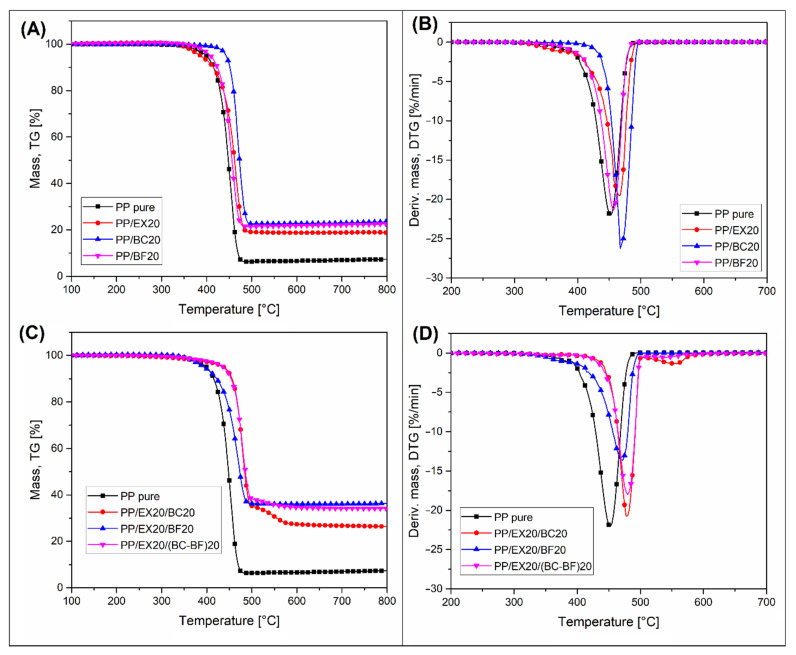
The thermogravimetric (TGA) analysis of PP-based samples. Results are presented in the form of weight loss TG (**A**,**C**) and derivative weight loss plots DTG (**B**,**D**).

The results of the TGA analysis measurements are presented in the form of thermograms of weight loss (TG) and its first derivative (DTG). The plots for the input materials, pure polymers, and fillers are presented in Figure 3. Analogous thermograms for composite samples are presented in the graphs in Figure 4 and Figure 5, respectively, for PLA and PP-based samples. Additional data (DTG peak position and char residue) are reported in Table S1 (Supplementary Information Section).

The analysis of the results of the pure matrix materials clearly shows a significantly higher thermal stability for PP. The weight loss caused by polymer decomposition of PP occurs at a temperature of about 400 °C, while for PLA, this process begins at around 350 °C. Similar results can be seen in the DTG curves where the shift of the PP and PLA peaks is about 90 °C in favor of PP. Due to the lack of additives in the form of fillers and a low tendency of residual char formation, the final weight of the PP and PLA samples is close to zero.

The TG curve for the EX flame retardant agent indicates a two-stage process of decomposition of this compound. The first signs of decomposition in the form of visible weight loss are already observed at 300 °C, which is confirmed by the peak in the DTG curve at 340 °C. At higher temperatures, the decomposition process stabilizes to regain intensity above 500 °C. The temperature of the second DTG peak is 610 °C. The active compound used in the used variant of the additive (EXOLIT AP 422) is ammonium polyphosphate (APP) [62,63,64]. The first observed degradation step, ending at a temperature of about 450 °C, is related to the decomposition of APP into ammonia and phosphoric acid. The mass loss then does not exceed 20%; however, the high temperature allows the cross-linking reaction to occur and the formation of polyphosphoric acid, causing the formation of a char layer. In the second stage of decomposition, occurring at a temperature of 500–700 °C, polyphosphoric acid is decomposed into phosphorus oxides forming a stable char. At 800 °C, the weight of the sample is reduced to 20%.

In the case of the other used filler, basalt fibers (BF), very high-temperature stability can be observed in the entire tested measuring range. The initial weight of the sample was not changed even at 800 °C. The observed loss of 2% is related to the decomposition of the fiber sizing agent.

Biocarbon (BC) filler is characterized by a lower temperature stability. A weight loss of about 2% was already visible at the temperature of 100 °C; in the discussed case, it is related to the release of water vapor from the porous BC structure. A further stable weight loss, at the level of 15% at 800 °C, is due to the breakdown of the organic functional groups present in the BC structure. These are mainly the hydroxyl and carboxyl group residues of organic compounds that were decomposed during the biomass pyrolysis process. Interestingly, the presence of these compounds has, in many cases, a positive effect on the occurrence of increased adhesion at the polymer-filler interface BC [46,49,65].

In the case of the TGA analysis results for PLA-based composites (Figure 4), the thermal stability of the matrix is almost identical for all samples. The start of decomposition for composite samples coincides with the decomposition temperature of pure PLA. There is a slight deviation for the PLA/BC20 sample, where the onset of decomposition occurs at a temperature of about 10 °C lower than other materials. The reason for this phenomenon may be a partial degradation of the PLA structure during the processing procedure (extrusion and injection molding) caused by the moisture contained in the BC filler. Apart from a slight decrease in the PLA thermal stability of the sample from BC, it is worth noting that, for most of the samples, the residual char mass was very close to the filler content. This confirms the complete decomposition of the polymer matrix at high temperatures. Interestingly, for PLA/EX20 and PLA/EX20/BC20 samples, the presence of the second stage of weight loss (at 500 °C) confirmed the two-step mechanism of APP decomposition.

TGA measurements for PP samples are presented in Figure 5. Generally, the decomposition temperature of PP-based materials was significantly higher, and there is also a visible difference between the particular composites. The initial weight loss for most of the samples was similar to pure PP, which can be confirmed by comparing the temperature of a 5% weight loss. However, the addition of BC particles increased the thermal stability of composites by around 30 °C. This is an inverse change to that observed for the PLA samples. Similar to PLA, the residual char content for PP-based composites was very close to the initial content of the fillers. The only noticeable difference was observed for the PP/EX20/BC20 sample, where the EX filler decomposition was observed at 500 °C.

It is worth noting that, considering the general tendency of changes in thermal stability (5% weight loss), the addition of fillers to PLA does not significantly affect the decomposition temperature of the material, while for PP composites the decomposition temperature was slightly shifted. Similar results were obtained for the studies performed by Kadola et al. where PLA and PP-based samples were modified with three different fire retardants [66]. The introduction of modifiers to PP has always resulted in a significant change in the degradation temperature of the material, while the changes for PLA were relatively small. 

The thermogravimetry results cannot be considered a valuable method for flame retardancy analysis; however, for selected samples containing the EX filler, it is possible to observe the additional weight loss step associated with the decomposition of polyphosphoric acid. 

### 3.3. Thermal Properties, Phase Transitions—DSC Analysis

**Figure 6:**
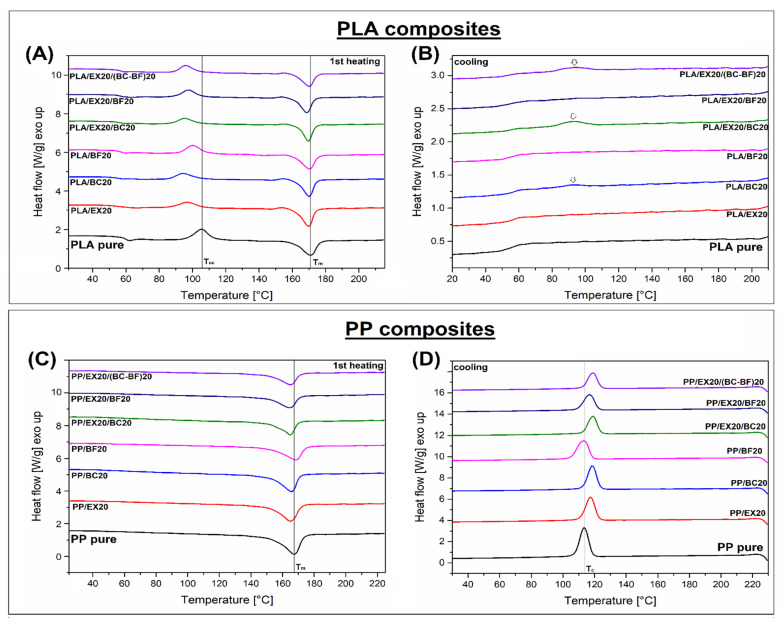
The DSC thermograms of (**A**,**B**) PLA and (**C**,**D**) PP-based materials. Plots present the 1st heating and cooling signals.

The results of the DSC thermal analysis are presented in the form of heating/cooling thermograms (see Figure 6). Some data and calculations are also presented in Table S2 (Supplementary Information Section). Since the melting temperature of PLA and PP are very close, the measurements were carried out in the same temperature range from room temperature up to 230 °C. The 1st heating signal for PLA-based samples (Figure 6A) and PP materials (Figure 6C) confirmed large differences in the phase transitions of the examined polymers. For PLA samples, the appearance of the cold crystallization phenomenon revealed a dominated amorphous nature of the matrix phase after the injection molding process. The presence of an increasing amount of the filler led to a small shift of the Tcc to a lower temperature (≈10 °C); however, the magnitude of that change is not significant. The melting temperature for all materials is close to 170 °C. Compared to PLA, the DSC analysis of PP-based samples revealed a less complex shape of the thermograms, while for all samples, only a single melting peak was recorded. Similar to PLA materials, the peak position for PP samples was almost equal for all measurements (≈165 °C). It seems that the addition of filler, even at a high amount of 40 wt%, did not lead to any significant change in composite characteristics during the heating stage of DSC analysis. The lack of differences between the plots’ appearance was confirmed by the crystallinity calculations (see Table S2). For PLA samples, the reference results for pure polymer revealed a 10% crystallinity level, while for most of the composites, the calculations show around 20% of the crystalline phase. The calculations for PP-based samples revealed that even for pure PP the crystallinity reached around 49%, which means that the main expected differences in thermomechanical properties between PLA and PP-based materials are caused by the crystallinity level. The DSC cooling stage for PP-based samples did not reveal any unpredictable changes. The crystallization temperature for composites was mostly slightly higher compared to pure PP. However, the magnitude of the temperature shift was small and cannot be compared to highly efficient nucleating agents such as sorbitol-based modifiers [67,68].

Interestingly, for both PLA and PP-based materials, the addition of BF led to a visible decrease in crystallinity. This behavior was recorded for PLA/BF20 and PP/BF20 samples; however, the hindering of the crystal phase growth at the observed level should not influence the important properties of composites. The described phenomenon, consisting of the reduction in the nucleation effect in PP as a result of the use of basalt-based materials, has already been observed in the literature [69]. Clearly, a more favorable nucleating effect was found for the BC additive, especially for PLA-based materials. For most of the tested materials, a cooling stage of the measurement did not reveal the distinct crystallization peak; it occurs only for samples containing BC particles. Unfortunately, the scale of this phenomenon is negligible, and it does not affect the level of crystallinity of the PLA/BC composites. However, it may be a suggestion for further work using PLA with a higher crystallization ability. 

The results of the DSC analysis revealed some visible changes in crystallinity for the PLA samples. However, despite the fact that for most composites, the content of the crystalline phase was two times higher than for pure PLA, the amorphous phase that still predominates in the matrix structure significantly contributes to the reduction in thermal resistance. Due to the fact that, for PP, the content of the crystalline phase is significant (≈50%), the presence of fillers does not contribute to any noticeable changes in phase structure. The addition of fillers may contribute to some changes in crystalline structure morphology, however, which might improve the mechanical properties of PP composites.

### 3.4. Mechanical Properties Evaluation—Static and Impact Tests

**Figure 7:**
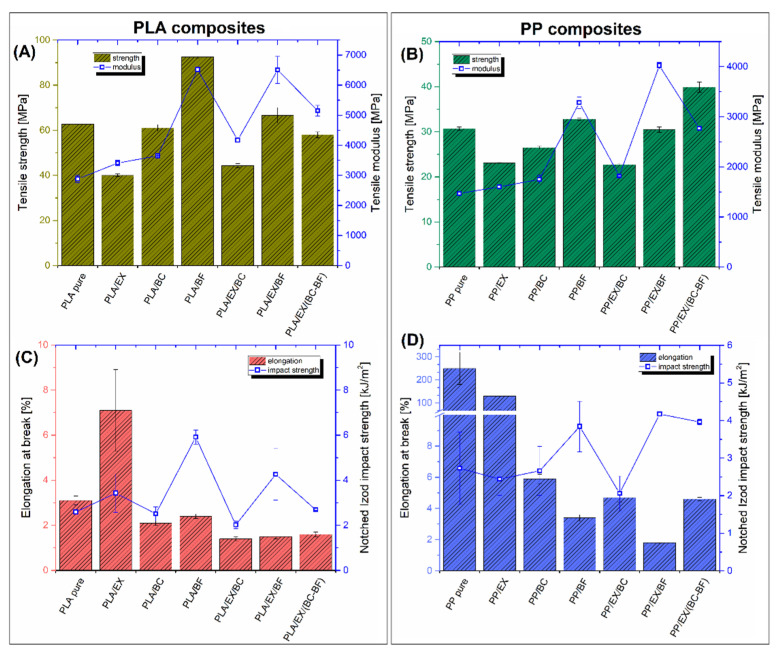
The results of the static tensile and Izod impact resistance tests. The tensile strength and modulus of (**A**) PLA-based and (**B**) PP-based composites. Elongation at break and impact strength, respectively, of (**C**) PLA and (**D**) PP-based materials.

The mechanical properties of the prepared materials are collected in the form of plots in Figure 7. The charts present the results of tensile strength/modulus, elongation at break, and notched Izod impact strength. The complete list of results is shown in Table S3 (Supplementary Information Section).

The results for PLA-based samples are presented in Figure 7A,C. It is clear that the addition of 20 and 40 wt% of the fillers increases the stiffness of the material (tensile modulus). However, the reinforcing efficiency was very different when considering the absolute values of the modulus. For pure powder fillers (EX and BC), the stiffness improvement was only slight, which was also observed for PP-based samples (see Figure 7B). The most visible improvement was recorded for the 20 wt% BF samples, where the PLA/BF sample modulus reached around 6.5 GPa, compared to the initial 3 Gpa, for pure PLA. The reinforcing effect for the PP/BF sample was also significant since the modulus was 3.3 Gpa, compared to the reference 1.5 GPa. For hybrid composites, the total content of the fillers was 40 wt%. However, the introduction of the EX/BC system did not improve the stiffness since the tensile modulus values were very close to the results of the BC-modified samples. This tendency was similar for PLA and PP-based samples as well. More favorable results were obtained for the hybrid samples containing reinforcing fibers. In comparison, the highest tensile modulus was recorded for EX/BF samples. Interesting conclusions can thus be made after the comparison of the tensile strength results.

For PLA-based composites, only the PLA/BF sample strength was visibly improved, up to 90 MPa from the initial 60 MPa for pure PLA. The lowest strength was recorded for the PLA/EX and PLA/EX/BC samples (≈40 MPa). This strength deterioration is quite an obvious phenomenon since the reinforcing efficiency for powdery fillers is low. For the other materials, the tensile strength results were close to the properties of pure PLA, from 60 to 70 MPa. Interestingly, for the PLA/BC samples, the measured strength was similar to pure PLA, which suggests that the matrix-filler interactions for the BC filler are stronger than for the EX particles. The results of the tensile strength measurement for PP-based composites revealed quite similar tendencies. Similar to PLA composites, the tensile strength was reduced for all samples with the addition of powdery fillers. Interestingly, the highest strength was observed for the PP/EX/(BC-BF) sample, while for the other BF reinforced composites (PP/BF and PP/EX/BF), the strength values are very close to the reference pure PP (≈35 MPa).

The elongation at break for all PLA-based composites was very low and usually did not reach 3%. However, some unexpected results were observed for the PLA/EX composite since the maximum strain increased up to 5%. The reason behind this improvement is not clear. However, part of the research indicates the occurrence of plasticizing effects with the use of small amounts of APP in PLA [19,70,71]. The partial solubility of APP in the polymer matrix is due to the low molecular weight of this compound. The micromechanism that improves the maximum elongation is caused by the change in the distance between the matrix polymer chains, which leads to the reduction in internal stresses during deformation. Unfortunately, in the case of the discussed samples, the effectiveness of this phenomenon is very limited and incomparably lower than the changes observed for more effective plasticizers [72,73,74]. Unlike pure PLA, the initial elongation at break for pure PP can reach even 250%. In comparison, the addition of fillers leads to a sharp reduction in sample strain. For most of the composite samples, the fracture appeared below 6% strain. Again, the EX-filled samples were characterized by different behavior, while the elongation at break for PP/EX composites was around 120%.

The impact resistance was relatively low for all of the prepared samples; however, some favorable improvement was noticed for BF-reinforced samples. For PLA-based samples, the reference impact strength was 2.5 kJ/m^2^ (for pure PLA), while the highest value was recorded for PLA/BF samples, reaching 6 kJ/m^2^. The visible improvement was also observed for the PLA/EX/BF sample (4.5 kJ/m^2^), while for the rest of the composites, the impact strength was close to 3 kJ/m^2^ or lower. Similar trends were observed for PP-based samples, where, for all BF-reinforced samples, the impact strength was close to 4 kJ/m^2^. For the rest of the materials, the impact strength was below 3 kJ/m^2^. The results of the impact tests show a favorable change in the fracture mechanism for the samples with the BF addition. The fiber pull-out mechanism, the main phenomenon leading to the toughness improvement in composite materials, is strongly limited for injection molded samples [75,76]; however, the impact strength is still visibly higher compared to the pure polymer. For the standard manufacturing procedure, where staple fibers are added to the matrix during the melt mixing procedure, the final filler length rarely exceeds 1 mm. That leads to a reduced interaction efficiency at the fiber-matrix interface [77,78]. 

Summarizing the mechanical test results, it can be noted that the addition of fibrous fillers is necessary to obtain relatively good mechanical performance. Without the BF reinforcement, the composites’ brittleness led to mechanical property deterioration. 

### 3.5. Thermo-Mechanical Properties—Comparison between DMTA Analysis and HDT/Vicat Tests

**Figure 8:**
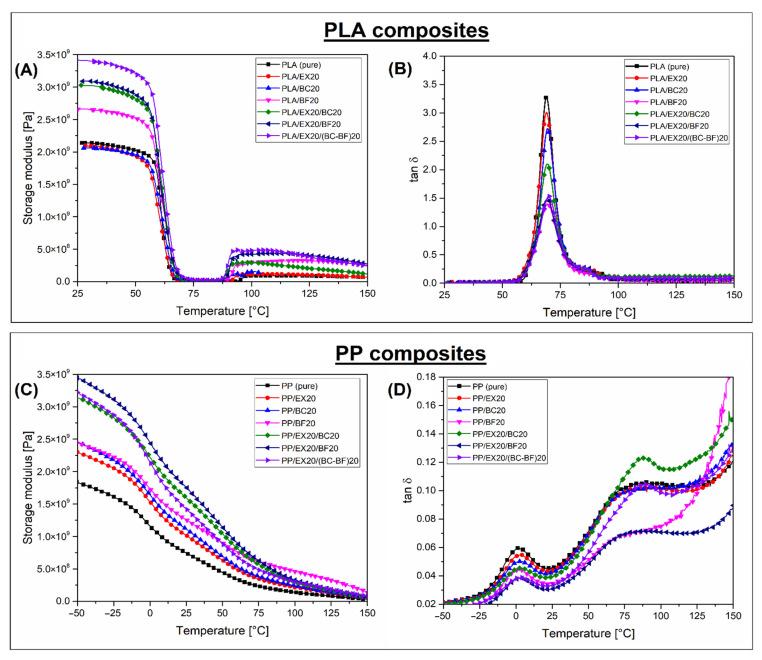
The results of the DMTA analysis of (**A**,**B**) PLA -based and (**C**,**D**) PP-based composites. The plots collect the results of the storage modulus and tan δ measurements.

**Figure 9:**
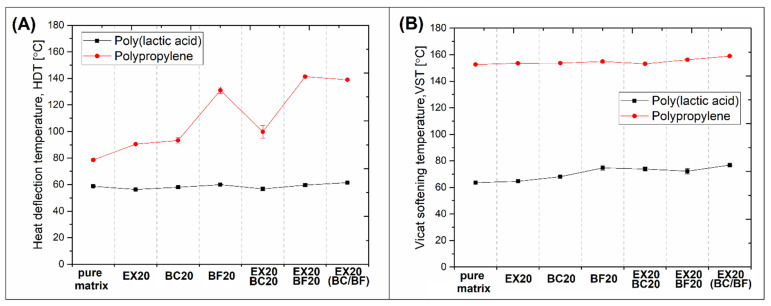
The results of (**A**) HDT and (**B**) VST measurements of all PLA and PP-based samples.

Since low thermal resistance is one of the most important limitations in the use of PLA, the thermomechanical properties of the prepared composites were investigated using DMTA analysis and a combination of heat deflection (HDT) and Vicat softening (VST) measurements. The results of DMTA tests are presented in the form of storage modulus and tan δ plots (see Figure 8). The HDT and VST test results are presented in Figure 9. The additional DMTA analysis for pure polymers is presented in Figure S1 (Supplementary Information Section).

The comparison of the storage modulus for PLA-based samples (see Figure 8A) revealed large differences in material stiffness for different types of fillers. It can be expected that the reinforcing efficiency of the powdery fillers was negligible since the storage modulus values for PLA/EX20 and PLA/BC20 composites are almost similar to pure PLA. Visible changes were recorded for PLA/BF20 samples, which confirmed a more favorable strengthening mechanism. The highest stiffness was observed for composites with the addition of 40 wt% of the fillers, while the highest storage modulus was recorded for the PLA/EX20(BC-BF)20 sample. Despite the application of a large amount of filler, the glass transition phenomenon led to a large drop in storage modulus values. For all samples, the sharp stiffness reduction observed at around 60 °C led to material softening, resulting in a significant reduction in the use at higher temperatures. The tan δ plots confirmed that the Tg region for all composites was constant, which confirmed that strong filler/matrix interactions did not occur. The only noticeable difference refers to the area under the Tc peak, where, for highly filled samples, the peak value was strongly reduced. That phenomenon is typical for composite samples since the presence of the filler particles leads to a reduction in the polymer volume in the sample [79,80]. The appearance of the DMTA plots for PLA-based samples confirmed the amorphous character of the matrix phase. At higher temperatures (>90 °C), the storage modulus values increase, which is related to the cold crystallization phenomenon. However, it confirmed that during the standard injection molding process, where the mold temperature is below 100 °C, the possibility of obtaining a highly crystalline form of PLA is not possible.

The DMTA analysis for PP-based composites differs significantly from the results for PLA, mostly due to the highly crystalline structure of the matrix phase. Since the T_g_ of PP usually occurs close to 0 °C, the measurements were conducted from −50 °C in order to cover the whole glass transition region. The initial high storage modulus values for PP-based samples were similar to those obtained for PLA composites. That is quite natural since, at the beginning of the test, both polymers are in a glassy state. However, it is worth noticing that the starting temperature for PP-based materials was 75 °C lower than for PLA. The stiffness of PP recorded at 25 °C dropped to half its initial value. Such a decrease can be considered significant; however, the reduction in the modulus value throughout the whole measurement is relatively slow. The presence of the crystalline phase improves the thermal stability of the PP matrix. It is particularly evident in the glass transition region, where, unlike PLA, the PP composites do not lose stiffness so rapidly. Interestingly, for PP-based materials, the increase in the storage modulus was reported even for powder fillers, such as EX or BC. It is typical for highly crystalline polymers, where spherulites are formed around the filler particles [81,82]. The highest stiffness was reported for hybrid composites, with a 40 wt% addition of the fillers. Unlike PLA-based samples, where stiffness for all samples sharply drops at around 60 °C for all materials, the modulus for PP composites at elevated temperature (>50 °C) strongly depends on the filler content and type. This is why the expected thermal resistance will be highest for BF-reinforced hybrid samples. Similar to PLA, the position of the Tg peak was not shifted, which suggests a lack of strong filler-matrix interactions.

DMTA analysis makes it possible to observe, in a very accurate way, any changes in the stiffness of polymer composite materials on the temperature scale, but in industrial practice, thermomechanical resistance is usually evaluated using HDT or Vicat tests. Due to significant differences in measurement methodology, the results of these tests cannot be compared, therefore, it is not possible to use them interchangeably. In both cases, however, they are an indicator of the possible application temperature of the tested materials. The HDT results presented in Figure 9A confirm a large difference between PLA and PP-based materials. Even for pure polymer samples, there is around a 20 °C difference since HDT reached 60 °C and 80 °C, respectively, for pure PLA and pure PP. The difference for composites is even higher. This is easy to evaluate since, for all PLA-based composites, HDT was constant and close to the reference pure PLA. It is clear that for PP composites, the introduction of spherical fillers (EX and BC) led to only a small enhancement of the thermal resistance, a HDT of around 80–90 °C. A more significant improvement was recorded for BF, where for the PP/BF20 sample, the HDT reached 130 °C, while for the hybrid samples, PP/EX20/BF20 and PP/EX20/(BC-BF)20, the heat deflection was around 140 °C. The most important fact revealed during the analysis of HDT results is the lack of visible HDT improvement for PLA-based composites, even for highly filled hybrid samples. The advantage of using PP-based materials is even more visible when analyzing the VST results (see Figure 8B). The softening temperature for all PP materials was above 150 °C, which is very close to the melting point of the PP matrix. Unlike the HDT measurements, where, for all PLA-based samples, the results were similar, the VST measurements revealed some noticeable improvement after the addition of the fillers: from an initial 64 °C for pure PLA up to 77 °C for the PLA/EX20/(BC-BF)20 sample. However, that kind of difference cannot be considered a serious advantage. In such a situation, the beneficial role of the crystalline phase is revealed. Previous studies in this area confirmed that PLA-based composites are able to withstand prolonged exposure to high temperatures. However, the investigated samples were usually prepared by compression molding or annealing [83,84].

A thermomechanical analysis revealed significant drawbacks for all PLA-based injection-molded materials. For PLA, like other thermoplastic polyesters, such as PET or PBT, the crystallization process is much slower than for other semi-crystalline polymers such as PP, PE, or PA6. The manufacturing of specialized products requires the use of a high mold temperature, which unfortunately always increases production costs and process efficiency, and it often involves the use of dedicated nucleation agents [79,85,86,87].

### 3.6. Flame Retardancy—PCFC Microcalorimetric Analysis, UL-94 Tests

**Figure 10:**
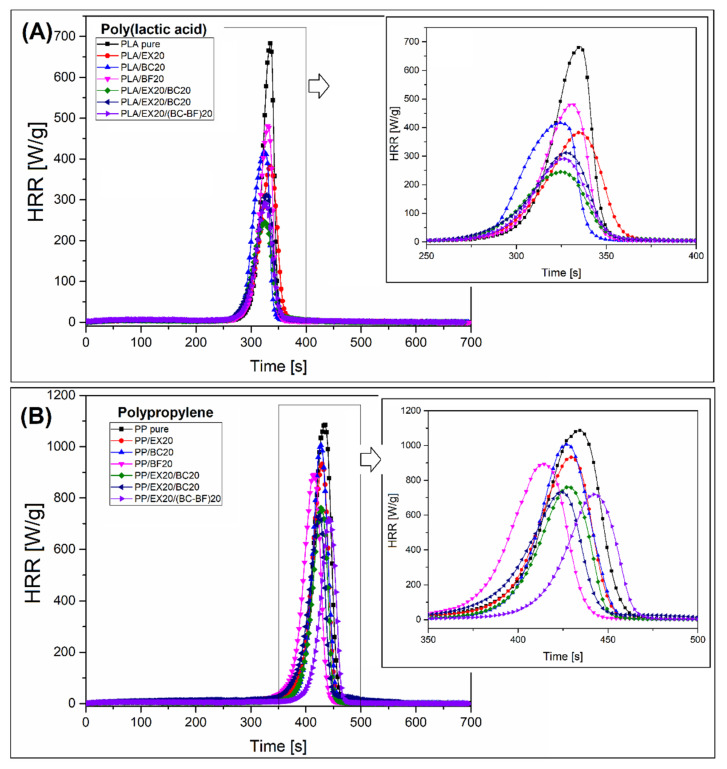
The relationship of the heat release rate (HRR) as a function of the measurement time of materials based on (**A**) PLA and (**B**) PP.

The results of the microcalorimetric analysis are collected in the form of separate plots for PLA-and PP-based samples in Figure 10. Some data and calculations are also presented in Table S4. The results of microcalorimetric measurements provide slightly more reliable information on the behavior of the tested materials during decomposition in the oxidizing environment, a distinction from TGA testing tests. The initial analysis of the HRR curve appearance indicates that the dynamics of the combustion process for most samples were very similar, which would suggest no significant differences in flammability. For other studies where phosphorous-based FR was used, the calorimetric measurements indicate a significant extension of the burning time of samples modified with APP at the same time the height of the HRR peak is reduced [66,71,88,89]. Regarding the PCFC measurements performed, the results are not conclusive and require additional calculations of the THR index, the changes of which slightly more clearly indicate the improvement of the results in the flammability tests.

It is clear that, for all samples, the heat release rate plots (HRR) revealed a single-stage process. Heat release is considered the most critical factor during the assessment of polymer composite flammability, while the position of the HRR curve peak is usually treated as the key factor during combustion calorimetric measurements. For PLA-based samples, it is clear that the HRR peak was highest for pure PLA samples, while the addition of the fillers led to a reduction in the HRR maximum. Among samples with the addition of 20 wt% fillers, the lowest HRR peak was observed for the PLA/EX20 sample; while comparing the hybrid samples, the lowest peak value was recorded for the PLA/EX20/BC20 sample. The results of the HRR peak height are in line with other factors calculated during the measurement. The results of the total heat release (THR) are very close to the HRR peak comparison where the lowest THR value was recorded for hybrid composites containing EX and BC particles. 

The HRR plots for PP composites revealed less efficient flammability reduction. Although the trend of HRR changes after the addition of fillers is favorable, the reduction in HRR and THR values indicates that materials’ flammability will be relatively high. Even for the PP/EX20/(BC-BF)20 sample, where THR was reduced to 26.7 kJ/g, the result was worse than for pure PLA (THR = 20 kJ/g). This difference gives a clear idea of the flame retardant potential for both types of material, whereas, even for highly modified PP samples, the flammability reduction will be less effective, which was confirmed by other studies [90]. The differences in the results of the microcalorimetric analysis were confirmed during the standard UL-94 tests. 

**Figure 11:**
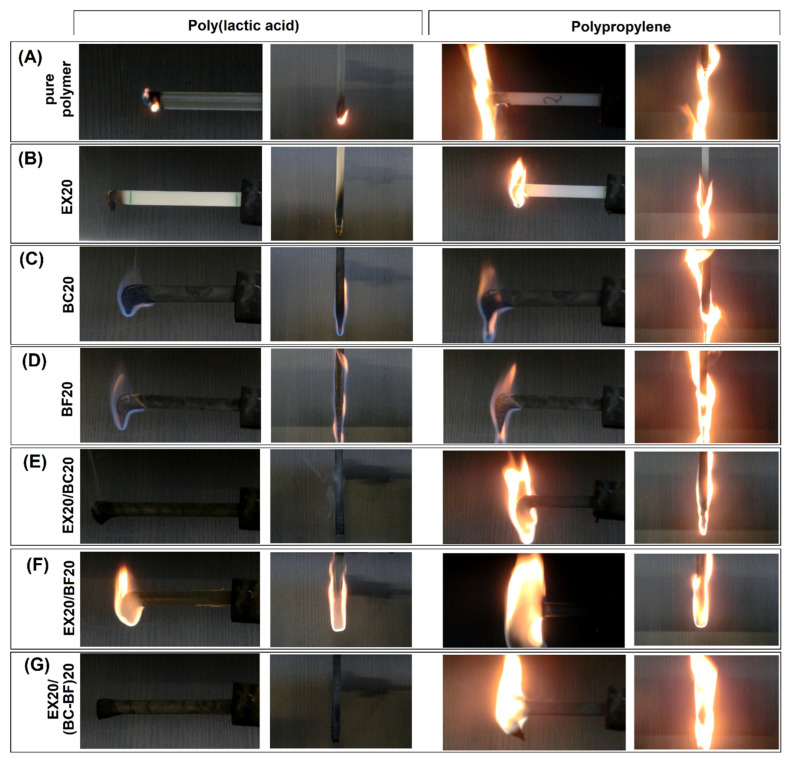
The sample appearance during the UL-94 tests (horizontal and vertical). The photos are grouped according to the type of fillers used.

The burning classification for the prepared materials was evaluated using the UL-94 testing methodology. All samples were tested in horizontal and vertical positions. The sample appearance during testing is presented in Figure 11 for both horizontal and vertical tests. The results of the performed tests are collected in Table 3.

The results for the PLA and PP-based samples are extremely different. The modification of PLA was evidently more favorable for PLA composites since, for selected composites, it was possible to obtain a V-0 flammability rating. It was possible for all of the prepared specimens to withstand the HB (horizontal burning) requirements since the highest burning speed recorded for pure PP was around 35 mm/min. For other samples, the burning speed varied from 16.7 to 32.6; however, there were no visible trends related to the sample composition. In general, the flammability of hybrid samples was lower. For PLA/EX20, PLA/EX20/BC20, and PLA/EX20/(BC-BF)20, the flame was rapidly self-extinguishing, while for the rest of the PLA-based materials, the tested specimens were completely burned. This behavior confirmed the importance of the APP addition, while the presence of polymer fillers, even at high quantities, did not improve flame retardancy. For PP-based samples, the results are less favorable since, for all specimens, the material burned along the whole sample length. The only visible improvement was observed for the PP/EX20/BF20 composite, where the material did not drip from the burning specimen. For other materials, the molten polymer dripped from the sample and ignited the cotton cloth placed under the specimen.

The results revealed that the addition of phosphorus-based APP flame retardants is more efficient for PLA-based samples. Interestingly, the obtained hybrid samples are characterized by V-0 class. This must be considered a very good result since the APP weight content in all samples was only 20%. The less favorable observed properties of the PP-based samples were mainly associated with a lack of oxygen present in the PP chain structure as well as the lower density of PP, which leads to a lower volumetric proportion of APP in the structure of the material. The lower volume of the flame-retardant additionally reduces its efficiency.

The vertical test results reflect the trends observed in the horizontal position. A self-extinguishing flame was observed again for the same three samples containing pure APP filler (EX20), EX20/BC20, and EX20(BC-BF)20 fillers. The remaining samples burned completely. It is clear that the presence of the EX additive is necessary to decrease the flammability of PLA, however, the use of fibrous reinforcement (BF) inhibits the flame retardancy mechanism. The use of hybrid systems with the addition of EX, BC, and BF seems to be the most effective in this approach since they help to maintain a low flammability rate, simultaneously improving the mechanical characteristics of the composite. Unfortunately, the results for the flammability tests of the PP samples confirmed a lack of improvement. All specimens were totally burned. Except for the PLA sample, all the PP samples melted intensely, initiating the cotton fabric ignition. 

The conducted research shows that the use of biocarbon without the addition of APP does not limit the flammability of the composite. However, unlike the addition of the BF filler, it seems that the use of BC supports the mechanism of phosphorus-based additives. Previous research indicates that, in the case of BF reinforcement, the mechanism of the burning process of composite materials is subject to unfavorable changes. The research conducted by Tang et al. [91] suggests that, for PP/BF materials, the thermal conductivity of composites is increased, which leads to the heating and decomposition of the material in a large volume. The consequence of this behavior is the inability to form a permanent char layer that could prevent the propagation of the thermal decomposition of the material. Unfortunately, there are no similar measurements for PLA-based samples. However, taking into account the results of the cited works, it can be stated with some certainty that for the fine dispersion of small BC particles, the thermal propagation to the inside of the material must be significantly limited in relation to the relatively long BF fibers. Despite the presence of BF fibers in the hybrid composite, the formation of the charred layer is facilitated by the presence of BC particles.

Summarizing the obtained results, it is worth noting that it is not clear what the main mechanism limiting the flammability of composites contacting the EX/BC system is. Taking into account the results of previous work by Wang et al. [56], the physical mechanism that reduces the flammability of materials with the addition of BC and the active FR is associated with the increased thermal conductivity of the composite. The increased heat transfer leads to faster decomposition of FR, which increases the efficiency of the APP compound. In our opinion, this mechanism is not decisive since most of the previous studies, where carbon fillers with much better thermal conductivity were used, revealed that the addition of carbon black, graphite, or graphene leads to the formation of a protective char layer [92,93]. Nevertheless, the discussion on this topic has only a theoretical character. The planned further research will include a more detailed analysis of a selected group of materials to determine a presumed mechanism of operation of systems containing biocarbon.

## 4. Conclusions

The conducted research confirmed that it is possible to combine the sustainable nature of polymer compounds with their flame retardancy effects. The reinforcing system containing BC particles and BF fibers seems to be an effective additive improving the mechanical properties of materials with the addition of APP flame retardant. As the results show, the addition of BC filler plays an important role in increasing the efficiency of EX operation, while the system containing EX and BF is characterized by increased flammability. Unfortunately, the beneficial effects of using the new filler system are observed only for PLA, while the flammability of PP is not improved. The current research related to the use of APP indicates that the most likely cause of the observed flammability differences is the higher efficiency of APP additive in materials containing oxygen in the chain structure. The obtained research results are very promising; therefore, as part of further experiments, it is planned to use a similar system for technical polymers, such as nylons or styrene polymers. 

## Figures and Tables

**Table 1:** The list of basic properties of the pure polymers and fillers.

Polymers				
	Density (g/cm^3^)	Melt Flow Rate (g/10 Min)	Tensile Strength (MPa)	Izod Impact Strength (J/m)
PLA	1.24	22	62	16
PP	0.9	12	34	25
Fillers				
	Density (g/cm^3^)	Particle size (µm)	Bulk density (g/cm^3^)	Decomposition temperature (°C)
EX	1.9	15	0.7	>275
	Density (g/cm^3^)	Particle size (µm)	Biomass type	Carbon content (%)
BC	1.5	1	Wood chips	65
	Density (g/cm^3^)	Diameter (µm)	Fiber length (mm)	Sizing
BF	2.7	13	6.2	Silane (0.4%)

**Table 2:** The list of sample designation and composite formulations.

	Matrix Polymer	Ammonium Polyphosphate (EX)	Biocarbon (BC)	Basalt Fiber (BF)
Sample	(wt%)	(wt%)	(wt%)	(wt%)
PLA	100	0	0	0
PLA/EX20	80	20	0	0
PLA/BC20	80	0	20	0
PLA/BF20	80	0	0	20
PLA/EX20/BC20	60	20	20	0
PLA/EX20/BF20	60	20	0	20
PLA/EX20/(BC-BF)20	60	20	10	10
PP	100	0	0	0
PP/EX20	80	20	0	0
PP/BC20	80	0	20	0
PP/BF20	80	0	0	20
PP/EX20/BC20	60	20	20	0
PP/EX20/BF20	60	20	0	20
PP/EX20/(BC-BF)20	60	20	10	10

**Table 3:** The results of UL-94 tests performed in horizontal and vertical positions.

Sample	Horizontal Measurement		Vertical Measurement			Rating
	Burning Speed (mm/min)	Dripping	T1 * (s)	T2 * (s)	Dripping	
**PLA-based composites**						
PLA pure	21.4	Yes	10	Total burn	Yes	HB
PLA/EX20	SE *	Yes	0	0	No	V0
PLA/BC20	32.2	Yes	Total burn	-	Yes	HB
PLA/BF20	32.6	Yes	Total burn	-	Yes	HB
PLA/EX20/BC20	SE	No	0	0	No	V0
PLA/EX20/BF20	25.9	Yes	Total burn	-	No	HB
PLA/EX20/(BC-BF)20	SE	No	0	3	No	V0
**PP-based composites**						
PP pure	34.8	Yes	Total burn	-	Yes	HB
PP/EX20	22.1	Yes	3	Total burn	Yes	HB
PP/BC20	25.9	Yes	Total burn	-	Yes	HB
PP/BF20	27.7	Yes	Total burn	-	Yes	HB
PP/EX20/BC20	18.8	Yes	5	Total burn	Yes	HB
PP/EX20/BF20	27.3	No	Total burn	-	No	HB
PP/EX20/(BC-BF)20	16.7	Yes	Total burn	-	Yes	HB

* T1/T2—burning time after 1st and 2nd ignition; * SE—self-extinguishing.

## Data Availability

Not applicable.

## Supplementary Materials

The following supporting information can be downloaded at: https://www.mdpi.com/article/10.3390/polym14194086/s1, Figure S1: The storage modulus/tan δ plots for (A) pure PLA and (B) pure PP sample; Table S1: The basic data collected during the thermogravimetric (TGA) measurements; Table S2: The results of DSC measurements from 1st heating and cooling stage; Table S3: The list of mechanical properties obtained during the static tensile/flexural measurements and Izod impact tests; Table S4: Results obtained during testing of PLA and PP-based composites using a PCFC microcalorimeter.
