# Supplementary material for: Development of a New Type of Flame Retarded Biocomposite Reinforced with a Biocarbon/Basalt Fiber System: A Comparative Study between Poly(lactic Acid) and Polypropylene
Source: Polymers (Basel). 2022 Sep 29;14(19):4086. doi: 10.3390/polym14194086 (PMC9572391; doi:10.3390/polym14194086)
Supplement: Supplementary file 1 [file polymers-14-04086-s001.zip › polymers-1927834-supplementary.pdf]

<u>Supplementary information</u>

Development of the new type of flame retarded biocomposites reinforced with biocarbon/basalt fiber system. The comparative study between poly(lactic acid) and polypropylene

Jacek Andrzejewski [1,*], Sławonir Michałowski [2]

[1] Poznan University of Technology, Faculty of Mechanical Engineering, Institute of Materials Technology, Piotrowo 3 Stree PL-61-138 Poznan, Poland
[2] Faculty of Chemical Engineering and Technology, Cracow University of Technology, Warszawska 24, 31-155 Krakow, Poland
* Correspondence: jacek.andrzejewski@put.poznan.pl; Tel.: +48 61 665 5858 (optional; include country code; if there are multiple corresponding authors, add author initials)

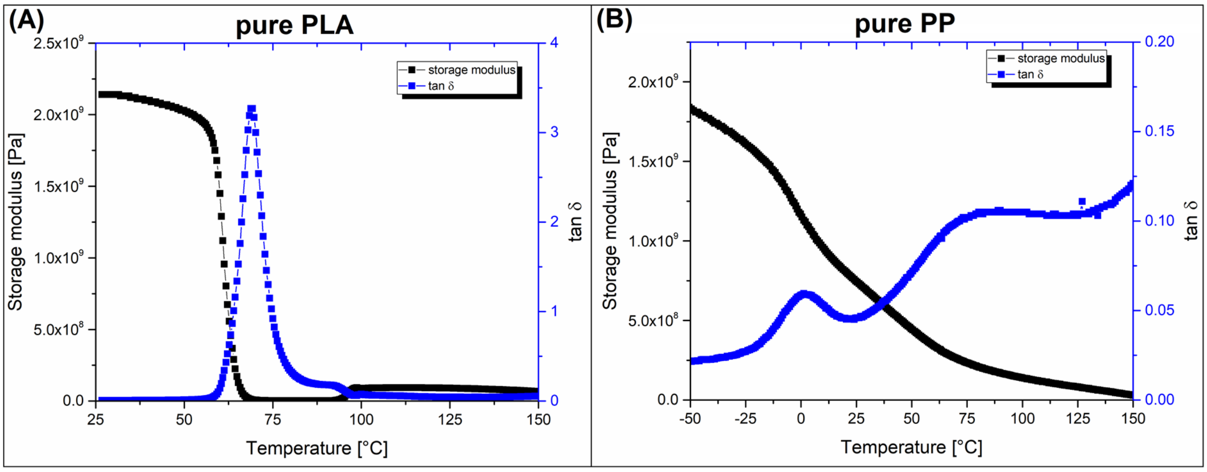

Figure S1. The storage modulus/tan δ plots for (A) pure PLA and (B) pure PP sample.

Table S1. The basic data collected during the thermogravimetric (TGA) measurements.

	$T_{5\%}$ [°C]	DTG_{peak1} [°C]	DTG_{peak2} [°C]	Residual char [%]
**PLA-based composites**				
PLA pure	325	362	-	2.4
PLA/EX20	330	360	540	10.5
PLA/BC20	317	345	-	20.9
PLA/BF20	328	360	-	19.8
PLA/EX20/BC20	327	350	495	31.8
PLA/EX20/BF20	327	355	-	33.0
PLA/EX20/(BC-BF)20	328	352	-	35.9
**PP-based composites**				
PP pure	400	452	-	7.3
PP/EX20	388	465	-	18.8
PP/BC20	443	470	-	23.4

PP/BF20	407	457	-	22.5
PP/EX20/BC20	437	480	550	26.3
PP/EX20/BF20	393	470	-	36.3
PP/EX20/(BC-BF)20	435	481	535	34.1

Table S2. The results of DSC measurements from 1st heating and cooling stage.

Sample	T_m peak [°C]	T_{cc} peak [°C]	T_c [°C]	H_{cc} [J/g]	H_m [J/g]	X_c [%]
**PLA-based samples**						
PLA	170.5	109.9	-	38.9	48.6	10.3
PLA/EX20	169.6	101.4	-	25.4	41.2	21.2
PLA/BC20	169.8	98.8	94.2	24.1	40.6	22.1
PLA/BF20	169.9	104.6	-	29.3	40.8	15.3
PLA/EX20/BC20	169.2	99.6	93.0	21.7	33.3	20.6
PLA/EX20/BF20	168.1	102.1	-	26.2	37.4	20.0
PLA/EX20/(BC-BF)20	169.8	100.2	94.5	22.6	32.5	17.6
**PP-based samples**						
PP	167.6	-	113.4	-	101	48.8
PP/EX20	165.0	-	117.3	-	84,4	51.0
PP/BC20	165.7	-	118.6	-	82.4	49.8
PP/BF20	168.6	-	113.3	-	73.1	44.1
PP/EX20/BC20	164.9	-	119.1	-	60.7	48.9
PP/EX20/BF20	164.8	-	116.9	-	61.8	49.8
PP/EX20/(BC-BF)20	165.1	-	119.1	-	59.3	47.8

Table S3. The list of mechanical properties obtained during the static tensile/flexural measurements and Izod impact tests.

	Tensile test			Flexural test		Izod test
	Modulus [MPa]	Strength [MPa]	Elongation at break [%]	Modulus [MPa]	Strength [MPa]	Impact strength [kJ/m^2]
**PLA-based samples**						
PLA pure	2880 (±124)	62.7 (±0.1)	3.1 (±0.2)	3560 (±62)	112.0 (±0.1)	2.6 (±0.3)
PLA/EX20	3410 (±94)	40.0 (±0.6)	7.1 (±1.8)	3580 (±63)	77.8 (±0.7)	3.4 (±0.8)
PLA/BC20	3650 (±7)	61.0 (±1.5)	2.1 (±0.1)	3780 (±75)	85.5 (±1.9)	2.5 (±0.3)
PLA/BF20	6516 (±75)	92.5 (±0.5)	2.4 (±0.1)	6630 (±200)	99.4 (±1.2)	5.9 (±0.3)
PLA/EX20/BC20	4170 (±116)	44.3 (±0.9)	1.4 (±0.1)	5100 (±129)	80.1 (±1.8)	2.0 (±0.2)
PLA/EX20/BF20	6507 (±447)	66.6 (±3.5)	1.5 (±0.1)	6630 (±200)	99.4 (±1.2)	4.3 (±1.1)
PLA/EX20(BC-BF)20	5150 (±179)	58.0 (±1.3)	1.6 (±0.1)	6400 (±85)	93.0 (±2.8)	2.7 (±0.1)
**PP-based samples**						
PP pure	1470 (±27)	30.7 (±0.4)	250.0 (±70.0)	1030 (±41)	30.6 (±0.3)	2.7 (±0.9)
PP/EX20	1600 (±29)	23.1 (±0.1)	130.0 (±60.0)	1440 (±30)	34.2 (±0.3)	2.4 (±0.4)

PP/BC20	1750 (±85)	26.5 (±0.3)	5.9 (±0.3)	1660 (±29)	41.8 (±0.3)	2.6 (±0.6)
PP/BF20	3280 (±113)	32.8 (±0.2)	3.4 (±0.2)	3030 (±132)	50.5 (±0.6)	3.8 (±0.7)
PP/EX20/BC20	1820 (±16)	22.7 (±0.3)	4.7 (±0.3)	2440 (±101)	46.2 (±1.6)	2.1 (±0.5)
PP/EX20/BF20	4020 (±65)	30.5 (±0.6)	1.8 (±0.1)	3930 (±216)	50.2 (±1.1)	4.2 (±0.6)
PP/EX20(BC-BF)20	2770 (±26)	39.9 (±1.1)	4.6 (±0.1)	3270 (±86)	66.8 (±1.2)	4.0 (±0.1)

Table S4. Results obtained during testing of PLA and PP-based composites using a PCFC microcalorimeter.

	pHRR [W/g]	T_{pHRR} [°C]	t_{pHRR} [s]	HRC [J/g·K]	THR [kJ/g]
**PLA-based composites**					
PLA pure	619 ±90	980 ±8	337 ±3	672 ±105	20.0 ±0.1
PLA/EX20	385 ±3	381 ±1	330 ±6	412 ±1	16.8 ±0.4
PLA/BC20	412 ±5	363 ±4	318 ±6	448 ±6	15.8 ±0.1
PLA/BF20	476 ±8	372 ±4	330 ±1	519 ±11	16.0 ±0.1
PLA/EX20/BC20	250 ±9	372 ±1	321 ±2	269 ±9	12.1 ±0.2
PLA/EX20/BF20	311 ±1	380 ±1	332 ±8	335 ±20	14.1 ±0.1
PLA/EX20/(BC-BF)20	290 ±7	374 ±1	328 ±5	315 ±7	12.4 ±0.8
**PP-based composites**					
PP pure	1110 ±35	472 ±1	425 ±13	1208 ±13	45.8 ±0.4
PP/EX20	653 ±28	479 ±1	438 ±12	1046 ±48	37.4 ±0.4
PP/BC20	1024 ±25	481 ±1	435 ±8	1116 ±37	36.9 ±0.8
PP/BF20	920 ±39	472 ±1	423 ±14	1000 ±61	37.6 ±0.3
PP/EX20/BC20	745 ±24	487 ±1	433 ±8	803 ±17	28.4 ±0.4
PP/EX20/BF20	739 ±9	481 ±1	430 ±8	801 ±20	34.0 ±0.4
PP/EX20/(BC-BF)20	695 ±35	487 ±1	443 ±1	761 ±38	26.7 ±0.6
